# Impact of the COVID-19 Pandemic on Physical Activity among Mostly Older, Overweight Black Women Living in the Rural Alabama Black Belt

**DOI:** 10.3390/ijerph20247180

**Published:** 2023-12-15

**Authors:** Whitney N. Neal, Erica A. Schleicher, Kerri Baron, Robert A. Oster, Nashira I. Brown, Wendy Demark-Wahnefried, Maria Pisu, Monica L. Baskin, Kelsey B. Parrish, William Walker Cole, Mohanraj Thirumalai, Dori W. Pekmezi

**Affiliations:** 1 Department of Health Behavior, University of Alabama at Birmingham, Birmingham, AL 35294, USA; eas33@uab.edu (E.A.S.); kelseyp@uab.edu (K.B.P.); colew14@uab.edu (W.W.C.); dpekmezi@uab.edu (D.W.P.); 2 O’Neal Comprehensive Cancer Center, University of Alabama at Birmingham, Birmingham, AL 35294, USA; roster@uabmc.edu (R.A.O.); mpisu@uabmc.edu (M.P.); 3 Capstone College of Nursing, University of Alabama, Tuscaloosa, AL 35487, USA; kabarron@ua.edu; 4 Department of Medicine, Division of Preventive Medicine, University of Alabama at Birmingham, Birmingham, AL 35294, USA; demark@uab.edu; 5 Department of Health Outcomes and Behavior, Moffitt Cancer Center, Tampa, FL 33612, USA; nashira.brown@moffitt.org; 6 Department of Nutrition Sciences, University of Alabama at Birmingham, Birmingham, AL 35294, USA; 7 Department of Medicine, Division of Hematology/Oncology, University of Pittsburgh, Pittsburgh, PA 15260, USA; baskinml@upmc.edu; 8 Health Services Administration, University of Alabama at Birmingham, Birmingham, AL 35294, USA; mohanraj@uab.edu

**Keywords:** coronavirus, exercise, physical activity, health disparities, rural health

## Abstract

Despite well-documented global declines in physical activity (PA) during the COVID-19 pandemic, little is known regarding the specific impact among underserved, rural Alabama counties. This is concerning as this region was already disproportionately burdened by inactivity and related chronic diseases and was among the hardest hit by COVID-19. Thus, the current study examined the effect of COVID-19 on PA in four rural Alabama counties. An ancillary survey was administered between March 2020 and August 2021 to the first cohort (N = 171) of participants enrolled in a larger PA trial. Main outcomes of this survey included the perceived impact of COVID-19 on PA, leisure-time PA, and social cognitive theory (SCT) constructs at 3 months. Almost half of the participants reported being less active during the pandemic (49.7%) and endorsed that COVID-19 made PA more difficult (47.4%), citing concerns such as getting sick from exercising outside of the home (70.4%) and discomfort wearing a face mask while exercising (58%). Perceived COVID-19 impact on PA was significantly associated with education, household dependents, and gender (*p*’s < 0.05). More women, parents, and college graduates reported that the COVID-19 pandemic made PA more difficult. Overall, there were no significant associations between PA, SCT constructs, or perceived COVID-19 impact on PA scores at 3 months. While the pandemic made PA difficult for many participants, these barriers were not associated with leisure-time PA levels or related theoretical mechanisms of action, which bodes well for the success of our ongoing intervention efforts and the resiliency of these communities.

## 1. Introduction

The COVID-19 pandemic amplified historically rooted health disparities [1], including those related to physical activity (PA) levels among adults living in rural communities [2,3]. Global and national data indicate substantial declines in PA during the pandemic [4]. Estimated rates of adherence with the Department of Health and Human Services (DHHS) PA guidelines (≥150 min of moderate-intensity or ≥75 min of vigorous-intensity aerobic PA per week) [5] decreased by 41% (moderate-to-vigorous PA, MVPA) and 42.2% (vigorous PA, VPA) [4]. Although decreases in adherence to PA were more prominent in other countries (i.e., Argentina, Brazil, Chile, and South Africa), notable declines in MVPA and VPA (~50 min per week) were observed in the United States (US) [4]. This trend towards inactivity represents a serious public health concern given the critical role PA plays in cardiorespiratory fitness [5], inflammatory responses [6], immune function [7], some cancers [8], and COVID-19-related health outcomes. Specifically, a recent meta-analysis [9] found better health outcomes due to COVID-19 infection (i.e., decreases in hospitalizations and deaths) among individuals that engaged in consistent or some PA compared to individuals who were consistently inactive. 

While many factors played a role in these declines in PA during the pandemic, confinement strategies (i.e., “stay-at-home” and “shelter-in-place” orders) implemented to help reduce the transmission of the virus restricted individuals’ ability to engage in sufficient levels of PA to maintain health [10]. These confinement strategies may have exacerbated existing health disparities in access to social and environmental supports for PA in the rural US, where physical inactivity has been a concern for years due to inadequate resources [11]. The first US-based study to examine COVID-19′s impact on meeting PA guidelines in rural and non-rural residents found that rural participants were significantly less physically active than their non-rural counterparts, and participants who reported lack of indoor space as a barrier to PA were 93% less likely to meet PA guidelines than those who did not indicate lack of indoor space as negatively impacting PA [12]. A separate cross-sectional study in rural western North Carolina [13] found that the closure of parks and recreation centers (71.9%), stay-at-home orders (55.7%), cancellation of recreational sports (48.8%), and fitness facility closings (44.9%) were the most commonly reported barriers to PA. However, data for both studies were collected from mostly white participants (95.7% and 96%, respectively) and may not be generalizable to other racial/ethnic groups. 

The specific impact of COVID-19 on PA levels in underserved, rural Alabama Black Belt counties has yet to be studied and warrants investigation. The Black Belt is known for its dark, rich soil and large Black/African American population, encompassing central and west-central Alabama [14]. This region is disproportionately burdened by inactivity and pre-existing comorbidities (e.g., high blood pressure, heart disease, cancer, and type 2 diabetes) [15]. Well-documented environmental barriers to PA (e.g., poor walkability and few playgrounds, trails, and recreational facilities) and gaps in programs and policies (e.g., requirements for sidewalks and bikeways in new infrastructure projects) exist in these counties [16,17] and may have been compounded by pandemic-related restrictions. Black Belt counties were among the hardest hit during the pandemic, with nearly 30% more COVID-19 deaths in 2020 than non-Black Belt counties in Alabama [18] Such statistics raise concerns regarding the potential exacerbation of existing health disparities and call for examination of the pandemic’s impact upon important health indicators, such as PA levels, in this region. 

Thus, in the current study, we assessed the perceived impact of COVID-19 on PA barriers and behaviors among rural Alabama Black Belt residents. These ancillary data were collected remotely at the height of the pandemic (March 2020–August 2021) at the time of 3-month follow-up from the first cohort of participants enrolled in a larger randomized controlled trial (RCT) of interactive voice response (IVR) system-supported PA phone counseling [the Deep South IVR-Supported Active Lifestyle (DIAL) intervention] [19]. We hypothesized that PA was negatively impacted by COVID-19 in these Black Belt counties, as in other communities [4], and sought to shed light on how (and for whom) the pandemic made being physically active difficult. Moreover, we explored the associations between perceptions of the impact of COVID-19 on PA and other variables assessed at this time point, including leisure-time PA and related social cognitive theory (SCT) constructs (walking self-efficacy, social support, planning, goal setting, outcome expectations, and enjoyment, which are considered early indicators of behavior change). In line with previous research evaluating the relationship between SCT constructs and pandemic-era PA behavior [20], we hypothesized that perceptions of the impact of COVID-19 on PA would be negatively associated with leisure-time PA and SCT constructs; that is, individuals experiencing pandemic-related PA barriers may feel less confident (or self-efficacious) in their ability to be physically active, lack a supportive social network for staying active, find it difficult to maintain healthy self-regulation behaviors, etc. The results presented herein will help guide ongoing and future efforts to address rural health disparities and offset the disruption in daily PA routines caused by a public health crisis.

## 2. Materials and Methods

### 2.1. Study Design

The current ancillary study involved cross-sectional analyses to assess the perceived impact of COVID-19 on PA levels in rural Alabama Black Belt communities. Data were collected between March 2020 and August 2021 from the first cohort of participants enrolled in the larger DIAL trial. All participants in the parent study were randomly assigned to either the DIAL intervention or waitlist control (WLC) arm. The DIAL intervention targeted key SCT constructs by providing PA trackers [pedometers (Accusplit, AX2790MV) and Fitbits (Inspire)] and regular IVR-automated phone counseling (i.e., daily calls in months 0–3 on PA-related self-efficacy, social support, goal setting, outcome expectations, and enjoyment). Participants in the WLC arm were encouraged to maintain their usual activity levels. Primary outcomes for the current study (COVID-19’s impact on PA, self-reported PA, related SCT constructs) were measured remotely at the same time point (3 months follow-up). The trial was approved by the University of Alabama at Birmingham Institutional Review Board, and all participants provided informed consent consistent with standards outlined in the Common Rule prior to completing assessments. The overall study design for the larger RCT was described in a prior report [19].

### 2.2. Study Population

The current study focused on a subsample of participants in Greene, Marengo, Dallas, and Sumter counties in Alabama who were enrolled in the RCT at the height of the pandemic (March 2020–August 2021) and completed measures of COVID-19’s impact on PA. Participants were recruited for the parent study utilizing flyers, word-of-mouth, and advertisements via local newspapers and then screened for eligibility by research staff. Eligibility criteria for the parent study included (1) ≥18 years of age, (2) resident in the participating rural counties, (3) insufficiently active (engaging in <60 min of MVPA per week), (4) able to speak and read English, and (5) willing to adhere to the study protocols. Participants were excluded from involvement in the parent study if they (1) planned to move outside of the participating rural counties in the next 18 months, (2) did not have access to a telephone, or (3) had any medical condition that might make participation in unsupervised PA unsafe (i.e., history of stroke, heart disease, or an orthopedic condition based on the Physical Activity Readiness Questionnaire [21]). For the parent RCT, of the 240 potential participants who contacted us, 214 participants met eligibility criteria, and 185 participants were enrolled. Of the 185 participants who were enrolled in the parent study, 171 participants had complete data and were included in this ancillary cross-sectional study.

### 2.3. Assessments

#### 2.3.1. The Impact of COVID-19 on Physical Activity

The survey items to measure perceptions of how the COVID-19 pandemic impacted PA were developed by the research team and focused on behavior and barriers. The behavior item was “How has the COVID-19 pandemic impacted your [PA]?” and included the following response options: more active, less active, or no change. Participants also completed barriers items (“Has the COVID-19 pandemic made it more difficult to be active?” with yes/no response options). If participants answered yes, they were asked “How has the COVID-19 pandemic made [PA] difficult?” and could select all applicable options listed (exercise/fitness facilities were closed/less accessible, concerned about getting sick from exercising outside of the home, discomfort with wearing a face mask while exercising, increased childcare responsibilities, increased work responsibilities, increased stress, increased financial strain, other).

#### 2.3.2. Physical Activity

The Godin Leisure-Time Exercise Questionnaire (GLTEQ) was administered given its ease of use and excellent reliability and validity [22]. The GLTEQ measures the frequency of strenuous, moderate, and light PA performed for periods of 10 min or more during the previous 7-day period. An updated version that includes frequency and average minutes of duration of exercise per week within intensity categories [strenuous, moderate (including strength training), and mild] was selected for the current study [22]. 

#### 2.3.3. Social Cognitive Theory Variables

PA-related SCT constructs were also assessed (Table 1). An abbreviated version of the Self-Efficacy for Walking Scale [5 items, α = 0.97–0.98 pre- and post-exercise, possible scores ranging from 0% (not at all confident) to 100% (extremely confident)] [23] was used to measure walking self-efficacy. Social support was measured using the validated short form version of the 13-item Social Support for Exercise scale [3 items, α = 0.75, possible scores ranging from 1 (no social support) to 5 (social support received very often)] [24]. Goal setting and planning were measured using the 10-item Exercise Goal Setting (EGS) and Exercise Planning (EPS) Scales [(α_(EGS)_ = 0.89; α_(EPS)_ = 0.87), possible scores ranging from 1 (does not describe) to 5 (describes completely)] [25]. Outcome expectations were measured with the 9-item Outcome Expectations for Exercise scale [(α = 0.89, possible scores range from 1 (low expectations) to 5 (high expectations)] [26]. Lastly, PA enjoyment was measured with the Physical Activity Enjoyment Scale (PACES) (5 items, α = 0.75, possible scores ranging from 0 to 48, scores ≥ 24 are interpreted as higher enjoyment while being physically active) [27].

### 2.4. Data Analysis

Descriptive statistics were obtained for the entire cohort and then separately by group (intervention group, WLC group; COVID-19 was a barrier to exercise, COVID-19 was not a barrier to exercise). Continuous variables were assessed for normality using box, stem-and-leaf, and normal probability plots and the Kolmogorov–Smirnov test. Only leisure-time PA deviated substantially from a normal distribution; as such, leisure-time PA was analyzed on its original scale (minutes/week) and then on a log_10_ transformed scale. Since the results from both sets of analyses were very similar, we report results using leisure-time PA on its original scale for ease of interpretation and for consistency with much of the previous literature. Given the small amount of missing data (<1%) and exploratory nature of our study, there were no adjustments for missing data during the analyses. That is, during the analyses, imputation was not performed for missing data, and all available data were used in the analyses. Statistical tests were two-sided and were performed using a significance level of 0.05. Statistical analyses were performed using SAS software (version 9.4; SAS Institute, Inc., Cary, NC, USA).

The chi-square test (or Fisher’s exact test, if needed) was used to compare proportions between COVID-19 groups [two groups: COVID-19 made PA difficult (yes, no); three groups: less active, more active, no change in PA due to pandemic]. The two-group *t*-test was used to compare means between the two COVID-19 groups [COVID-19 made PA difficult (yes, no)], and analysis of variance was used to compare means between the three COVID-19 groups (less active, more active, no change in PA due to pandemic). For the latter, when a statistically significant difference was found among the three group means, the Tukey–Kramer multiple comparisons test was used to determine which specific pairs of means were significantly different.

## 3. Results

### 3.1. Participant Characteristics

A total of 171 DIAL participants completed surveys on perceived COVID-19 impact on PA (Table 2). This sample consisted of mostly Black/African American (98.2%) females (90.6%). The mean BMI was 36.32 (SD = 7.93), which is in the obese range (≥30 kg/m^2^), and the mean age was 57.1 years (SD = 13.26). Fewer than half of participants reported household incomes ≥ USD 30,000 per year (43.5%), living with children (32.4%), current employment (44.4%), or college degrees (45%).

### 3.2. Impact of COVID-19 on Physical Activity

**Figure 1:**
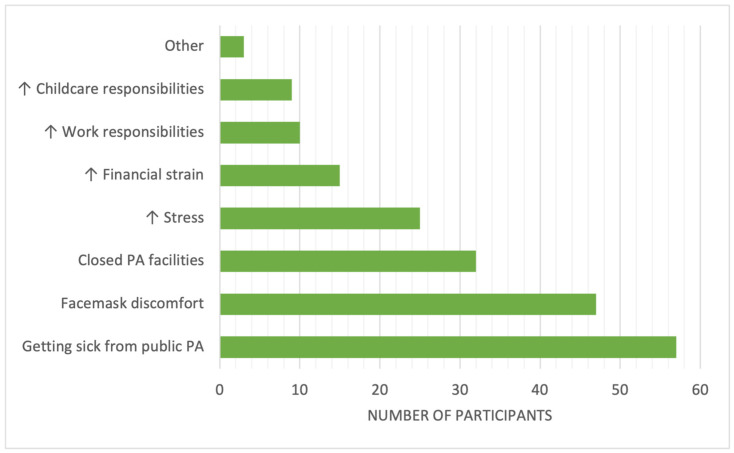
Concerns reported by participants who indicated the COVID-19 pandemic made it more difficult to be physically active (*n* = 81).

Roughly half of the participants (*n* = 85, 49.7%) reported being less active during the pandemic, whereas others reported being more active (*n* = 31, 18.1%) or no change in PA (*n* = 55, 32.2%). Overall, 47.4% of this sample (*n* = 81) endorsed that the COVID-19 pandemic made it more difficult to be active (*n* = 90, 52.6% did not), citing concerns such as getting sick from exercising outside of home (*n* = 57, 70.4%) and discomfort with wearing a face mask while exercising (*n* = 47, 58%), whereas others reported closed/less accessible exercise/fitness facilities (*n* = 32, 39.5%), stress (*n* = 25, 30.9%), financial strain (*n* = 15, 18.5%), work responsibilities (*n* = 10, 12.3%), and childcare responsibilities (*n* = 9, 11.1%) as barriers to PA engagement. Three participants who reported COVID-19 negatively impacting PA selected the “other” option and added “knee problems”, “caregiver for family member with COVID”, and “drained after experiencing COVID-19” (see Figure 1).

### 3.3. Differences in COVID-19’s Impact on Physical Activity by Demographic Characteristics

There were significant differences in perceived COVID-19 impact on PA (barriers) scores by gender, education, and household dependents (*p*’s < 0.05). More women (51%; vs. men, 12.5%), individuals with college degrees (57%; vs. without, 39.4%), and those with children living at home (58.2%; vs. no children at home, 41.7%) reported that the COVID-19 pandemic made it more difficult to be active (Table 3). 

Certain barriers may account for the differences between these groups. More participants with college degrees (39%) expressed concerns about getting sick from exercising outside of home than those without college degrees (28.7%). A higher percentage of parents with children living at home cited increased childcare responsibilities (21.9% vs. 4.2%), stress (37.5% vs. 27.1%), and financial strain (28.1% vs. 12.5%) than participants without children living at home. Only 2 men (of 16 total) reported that COVID-19 made PA difficult, and neither indicated experiencing increased childcare responsibilities or stress; in contrast, women did (11.4% and 31.6%, respectively). There were no significant differences in perceived COVID-19 impact on PA (barriers item) scores by county, employment status, marital status, annual household income, age, race, or BMI. Perceptions of change in PA due to COVID-19 (behavior item) scores also did not differ based on any of the previously mentioned demographic variables.

### 3.4. Differences in Leisure-Time PA and SCT Constructs by Perceived COVID-19 Impact on PA at 3 Months

Overall, there were no significant differences among self-reported PA, SCT constructs, and responses to the perceived COVID-19 impact on PA item (“Has the COVID-19 pandemic made it more difficult to be active?”). While significant differences were found between responses to the item on perceived change in PA behavior (more active/less active/no change) due to COVID-19 and leisure-time PA (*p* = 0.001) and SCT constructs (goal setting, outcome expectations; *p*’s = 0.042, 0.023, respectively), most were in the expected direction (Table 4). Specifically, participants (both arms) who reported being more active in response to the pandemic also reported significantly higher leisure-time PA, greater exercise goal-setting behaviors, and more positive outcome expectations compared to those who reported no change or less PA due to the pandemic (*p*’s < 0.05). There were no significant differences among the perceived change in PA response categories (more active/less active/no change) for the other SCT constructs (i.e., self-efficacy, planning, enjoyment, and social support).

### 3.5. Differences in Leisure-Time PA, SCT Constructs, and Perceived COVID-19 Impact on PA at 3 Months Based on Study Arm

There were no significant between-arm differences in leisure-time PA or SCT constructs based on perceived COVID-19 impact on PA (barriers item) or change in PA due to COVID-19 (behavior item). Similar results were found when examining within-arm differences, with one exception. In the WLC arm, PA planning scores were significantly higher among participants who reported that COVID-19 made being physically active difficult than those who did not indicate that COVID-19 negatively impacted PA (*p* = 0.041).

When examined by arm, the differences in self-reported PA between intervention participants who reported being more active, less active, and no change in PA in response to COVID-19 were statistically significant (*p* = 0.002), as those who reported being more active also reported significantly greater leisure-time PA than those who indicated they were less active in response to the pandemic (*p* < 0.001). However, there were no other significant associations between the perceived impact of COVID-19 on PA behavior and leisure-time PA and SCT constructs for the intervention and WLC arms (Table 5).

## 4. Discussion

These ancillary data analyses examined the impact of COVID-19 on PA in a rural, mostly Black/African American sample participating in a larger PA RCT between March 2020 and August 2021. Aligning with national and global trends [4], the findings point to a perceived negative impact of COVID-19 on PA participation—nearly half of participants described pandemic-related barriers that made PA more difficult and reported being less active during the height of the pandemic. Several of the pandemic-related barriers to PA (i.e., increased stress and anxiety, lack of facilities to partake in PA, fear of exposure to COVID-19, and discomfort wearing a face mask) found in these communities were similar to those reported in other studies [12,13,28] with different samples (mostly White, income > USD 60,000 [13], and not rural [12]). On the contrary, the other half of the study sample reported that the COVID-19 pandemic did not make it more difficult to be active. The impact of COVID-19 on PA items was assessed at 3 months. Thus, many of these participants had received substantial PA counseling at that timepoint, which may have influenced their perceptions.

Results from the current study indicated that the pandemic’s impact was far from homogeneous, as more women, parents, and well-educated individuals reported that COVID-19 made it particularly difficult to be active. Many factors may account for these differences. For example, women and parents with children living at home (highly overlapping categories) were more likely to endorse increased childcare responsibilities as a PA barrier during the pandemic. It is important to note that when the data for this study were collected, in-person instruction was the primary mode of learning for students in only one of the four counties; the other three counties relied on virtual or hybrid instruction for the 2020–2021 school year. This may explain the increased childcare responsibilities reported by participants with children living at home. Given that our findings are based on a small sample of male participants (*n* = 16), any gender differences found in the current study should be interpreted with caution.

Individuals with higher education levels expressed more concern about getting sick from exercising outside of the home than those with lower education. We note that although these results contradict a previous study linking higher socioeconomic status (SES, which includes education) with greater resources for coping with COVID-19 and less pandemic-related worry among Chinese participants [29], differences in findings may be due to the previous study combining education with other SES variables (i.e., income). When examined separately in the current study, higher education level may have translated to having a better understanding of viral transmission and potential high-risk situations (e.g., crowded gyms with questionable ventilation and/or mask requirements). This has been observed in past studies linking higher education levels to better COVID-19 knowledge, attitudes, and prevention practices [29,30,31], as well as reduced risk for belief in COVID-19 misinformation [32] and even COVID-19 infection, hospitalization, and mortality [33,34,35,36,37,38,39,40]. Clearer, more accessible health information may be critical for individuals with lower education levels in future crises, as those without a high school degree accounted for nearly one-fourth of COVID-19 deaths according to National Health and Nutrition Examination Survey (NHANES) data [35].

Despite the challenges identified (i.e., difficulty being physically active due to pandemic-related barriers such as increased stress and anxiety, less accessible facilities to partake in PA, fear of exposure to COVID-19, and discomfort wearing a face mask), our findings indicated that the perceived impact of COVID-19 on PA (barriers item) was not associated with leisure-time PA and related SCT constructs. This somewhat allays concerns about COVID-19 interference with our ongoing PA intervention efforts in the Black Belt. The findings suggesting higher PA planning scores among the WLC participants who reported pandemic-related PA difficulties (vs. those who did not) are intriguing. Perhaps these participants felt that PA participation amid such barriers required more effort and planning. While it is heartening that pandemic-related challenges were (for the most part) not associated with leisure-time PA and related SCT constructs, it is not surprising that the perceived change in PA due to COVID-19 (behavior item) scores were. Participants who reported being more active in response to COVID-19 also reported higher leisure-time PA, goal setting, and outcome expectation scores than those who reported being less active or no change in PA levels due to COVID-19. This emphasizes the potential importance of developing multicomponent interventions that provide information regarding expected outcomes or benefits of PA and incorporate self-regulation strategies, especially for participants pursuing behavior change on their own during a pandemic. 

The technology- and theory-supported strategies employed in the ongoing parent trial are well-suited for overcoming the barriers identified in these ancillary analyses (i.e., concerns about getting sick from exercising outside of home, discomfort with wearing a face mask while exercising, lack of access to fitness facilities, financial strain, work responsibilities, and childcare responsibilities). Home-based, self-directed approaches to increase PA engagement are low cost [41,42] and do not require participants to visit gyms or fitness facilities, interact with the public, or wear a mask while exercising, to name a few. Moreover, such interventions can be completed at the participant’s convenience (e.g., before/after work or while children are at school/napping). Other remotely delivered PA interventions have been used with success in various populations (i.e., older women living in the US [43], older adults with cognitive frailty living in Malaysia [44], and older adults with early dementia living in England, United Kingdom [45]) during the pandemic, which also bodes well for our efforts to address physical inactivity and related health disparities in the Black Belt.

### 4.1. Strengths and Limitations

This study is not without limitations. First, there was no follow-up to examine changes in perceptions about the impact of COVID-19 on PA participation as the pandemic persisted. Additionally, our population included mostly Black/African American adults living in rural Alabama; thus, findings may not generalize to other populations. Moreover, over 90% of participants were female; thus, male representation in our sample was not optimal (as in other community-based studies [12,13,46,47]). We also did not collect data from participants on COVID-19 infection status, and those who were infected with COVID-19 may have had different PA levels or priorities than participants who were not. Another potential limitation pertains to the reliance on self-reported data on leisure-time PA and several short-form psychosocial measures due to concerns for participant burden and safety during stay-at-home orders; nonetheless, we utilized validated surveys to assess leisure-time PA and psychosocial outcomes.

### 4.2. Future Directions

The results from this study present important practical implications. Top concerns related to PA participation reported by DIAL participants during the pandemic included getting sick from exercising outside of the home, inaccessible fitness facilities, and discomfort wearing a face mask while exercising. At the early stage of the pandemic, state and local restrictions resulted in the closure or limitation of public spaces, such as parks, outdoor walking trails, and fitness facilities; however, we now know that these closures resulted in adverse effects on PA engagement [48]. Now that more is known regarding the spread of such viruses, such excessive and harmful measures could be avoided in the future if public health agencies assist in raising awareness of available outdoor PA locations, such as local parks and trails, and educate residents on safely exercising in indoor fitness facilities. 

Following stay-at-home orders, modified reopening plans implemented during the pandemic, such as the Alabama Safer-at-Home order [49], required that a face mask be worn while exercising at gyms and fitness facilities. Although the physiological effects of wearing a face mask are minimal and unlikely to impact exercise performance among healthy individuals without cardiorespiratory disease [50], discomfort associated with wearing a face mask while exercising has been a commonly reported psychological barrier to PA during the COVID-19 pandemic [51]. Future research should focus on methods to reduce the negative perception of wearing a face mask while exercising, as these perceptions may adversely impact MVPA.

## 5. Conclusions

The study results indicate that nearly half of the rural, mostly minority participants from the Alabama Black Belt reported engaging in less PA during the pandemic and encountered challenges to being physically active due to COVID-19. Barriers were identified (i.e., concerns about getting sick from exercising outside of the home, discomfort with wearing a face mask while exercising, and inaccessible fitness facilities), along with groups from this region whose PA was disproportionately impacted by the pandemic (i.e., women, individuals with higher education levels, and those with children living at home). Fortunately, these difficulties were not associated with leisure-time PA or related SCT constructs, which suggests that the efficacy of the ongoing intervention may not be negatively influenced by pandemic-related challenges. In fact, the low-cost PA phone counseling approach used in the parent study appears ideal for addressing the barriers identified in the current study and promoting PA engagement in this underserved target population, even during a public health crisis.

## Figures and Tables

**Table 1:** Social cognitive theory battery of assessments.

SCT Construct	Tool
Self-efficacy	5-Item Self-Efficacy for Walking Scale [23]
Social support	3-Item Social Support for Exercise Scale [24]
Goal setting	10-Item Exercise Goal Setting Scale [25]
Planning	10-Item Exercise Planning Scale [25]
Outcome expectations	9-Item Outcome Expectations for Exercise Scale [26]
Enjoyment	18-Item Physical Activity Enjoyment Scale [27]

**Table 2:** Demographic characteristics of participants who completed surveys on perceived impact of the COVID-19 pandemic on physical activity (N = 171).

Variable	N (%)
County	
Marengo	41 (24%)
Dallas	42 (24.6%)
Greene	49 (28.6%)
Sumter	39 (22.8%)
Education level	
College degree	94 (55%)
No college degree	77 (45%)
Employment status	
Full/part time	76 (44.4%)
Unemployed ^1^	95 (55.6%)
Living with children ^2^	
Yes	55 (32.4%)
No	115 (67.6%)
Gender	
Male	16 (9.4%)
Female	155 (90.6%)
Marital status	
Married	61 (35.7%)
Not married	110 (64.3%)
Annual household income ^3^	
<$30,000	95 (56.5%)
≥$30,000	73 (43.5%)
Age ^4^	
≥60 years old	88 (51.5%)
<60 years old	83 (48.5%)
Race	
Black/African American	168 (98.2%)
White	3 (1.8%)
BMI ^5^	
≥30	130 (76.5%)
<30	40 (23.5%)
Study arm	
Intervention	84 (49.1%)
Waitlist control	87 (50.9%)

^1^ Unemployed includes retired, student, and disabled; ^2^ N = 170; ^3^ N = 168; ^4^ M = 57.12, SD = 13.26; ^5^ BMI = body mass index, N = 170, M = 36.32, SD = 7.93.

**Table 3:** Descriptive statistics of demographic characteristics of the sample split by perceived impact of the COVID-19 pandemic on physical activity.

Variable	Did the COVID-19 Pandemic Make PA More Difficult?		*p*-Value
	Yes (*n* = 81) *n* (%)	No (*n* = 90) *n* (%)	
County			
Marengo	17 (41.5%)	24 (58.5%)	0.080
Dallas	27 (64.3%)	15 (35.7%)	
Greene	22 (44.9%)	27 (55.1%)	
Sumter	15 (38.5%)	22 (61.5%)	
Education level			
College degree	37 (39.4%)	57 (60.6%)	0.021 ^1^
No college degree	44 (57.1%)	33 (42.9%)	
Employment status			
Full/part time	37 (48.7%)	39 (51.3%)	0.758
Unemployed	44 (46.3%)	51 (53.7%)	
Living with children ^1^			
Yes	32 (58.2%)	23 (41.8%)	0.045 ^1^
No	48 (41.7%)	67 (58.3%)	
Gender			
Male	2 (12.5%)	14 (87.5%)	0.003 ^2^
Female	79 (51%)	76 (49%)	
Marital status			
Married	29 (47.5%)	32 (52.5%)	0.973
Not married	52 (47.3%)	58 (52.7%)	
Annual household income			
<$30,000	42 (44.2%)	53 (55.8%)	0.313
≥$30,000	38 (52.1%)	35 (48%)	
Age			
≥60 years old	36 (40.9%)	52 (59.1%)	0.082
<60 years old	45 (54.2%)	38 (45.8%)	
Race ^3^			
Black/African American	80 (47.6%)	88 (52.4%)	
White	1 (33.3%)	2 (66.7%)	
BMI ^4^			
≥30	63 (48.5%)	67 (51.5%)	0.509
<30	17 (42.5%)	23 (57.5%)	
Study arm			
Intervention	41 (48.8%)	43 (51.2%)	0.711
Waitlist control	40 (46%)	47 (54%)	

^1^ Denotes statistical significance at *p* < 0.05; ^2^ denotes statistical significance at *p* < 0.01; ^3^ Statistical testing was not employed for race due to an insufficient sample size for White participants; ^4^ BMI = body mass index.

**Table 4:** Physical activity and social cognitive theory variable scores categorized by perceived change in physical activity due to the COVID-19 pandemic.

Variable	Less Active (*n* = 85)	More Active (*n* = 31)	No Change in PA (*n* = 55)	
	Mean (SD)	Mean (SD)	Mean (SD)	*p*-Value
Leisure-time PA (min/week)	90.6 (81.1)	177.0 (97)	111.3 (92.4)	<0.001 *
Social support ^1^	8.3 (2.7)	8.9 (3.7)	7.9 (3.3)	0.377
Outcome expectations ^2^	4.0 (0.7)	4.4 (0.5)	3.9 (0.9)	0.023 **
PA enjoyment ^3^	3.7 (0.9)	3.9 (0.9)	3.8 (0.9)	0.527
Goal setting ^4^	2.7 (1.1)	3.2 (1.1)	2.6 (1.3)	0.042 ***
Planning ^5^	2.7 (0.6)	2.9 (0.7)	2.7 (0.8)	0.346
Walking self-efficacy ^6^	52.8 (31.4)	64.5 (28.2)	53.3 (31.6)	0.174

^1^ Social Support for Exercise Scale, possible scores range from 3 (no social support) to 15 (social support received very often); ^2^ Outcome Expectations for Exercise Scale: 1 (low expectations) to 5 (high expectations); ^3^ Physical Activity Enjoyment Scale: 1–5 (low to high enjoyment while being physically active); ^4^ Exercise Goal Setting Scale, possible scores range from 1 (does not describe) to 5 (describes completely); ^5^ Exercise Planning Scale, possible scores range from 1 (does not describe) to 5 (describes completely); ^6^ Self-efficacy for Walking Scale, possible scores range from 0 (not at all confident) to 100 (extremely confident). * For leisure-time PA, the “less active” mean is significantly less than the “more active” mean (*p* < 0.05), and the “no change” mean is significantly less than the “more active” mean (*p* < 0.05). ** For outcome expectations, the “less active” mean is significantly less than the “more active” mean (*p* < 0.05), and the “no change” mean is significantly less than the “more active” mean (*p* < 0.05). *** For goal setting, the “no change” mean is significantly less than the “more active” mean (*p* < 0.05).

**Table 5:** Descriptive statistics of physical activity and social cognitive theory variable scores in the intervention and WLC groups categorized by perceived change in physical activity due to the COVID-19 pandemic.

	Intervention			Wait-List Control		
Variable	Less Active (*n* = 40)	More Active (*n* = 22)	No Change in PA (*n* = 22)	Less Active (*n* = 45)	More Active (*n* = 9)	No Change in PA (*n* = 33)
	Mean (SD)	Mean (SD)	Mean (SD)	Mean (SD)	Mean (SD)	Mean (SD)
Leisure-time PA (min/week)	133.5 (96.2) *^,+^	193.1 (105.3) *^,+^	111.3 (92.4) *	77.6 (85.3)	137.7 (61.2)	96.5 (88.3)
Social support ^1^	8.9 (3.4)	9.1 (3.5)	7.9 (3.3)	8.1 (2.2)	8.3 (4.4)	7.3 (3.1)
Outcome expectations ^2^	4.0 (1)	4.4 (0.5)	3.9 (0.9)	4.0 (0.8)	4.3 (0.4)	3.9 (0.8)
PA enjoyment ^3^	4.0 (0.8)	4.2 (0.7)	3.8 (0.9)	3.7 (0.9)	3.3 (1.1)	3.7 (0.9)
Goal setting ^4^	2.9 (1.3)	3.3 (0.9)	2.6 (1.3)	2.7 (1.1)	3.0 (1.4)	2.4 (1.2)
Planning ^5^	2.8 (0.9)	3.0 (0.7)	2.7 (0.8)	2.6 (0.7)	2.5 (0.7)	2.6 (0.8)
Walking self-efficacy ^6^	58.2 (32.8)	67.4 (26.3)	53.3 (31.6)	51.0 (31.7)	57.4 (33)	50.1 (30.8)

^1^ Social Support for Exercise Scale, possible scores range from 3 (no social support) to 15 (social support received very often); ^2^ Outcome Expectations for Exercise Scale: 1 (low expectations) to 5 (high expectations); ^3^ Physical Activity Enjoyment Scale: 1–5 (low to high enjoyment while being physically active); ^4^ Exercise Goal Setting Scale, possible scores range from 1 (does not describe) to 5 (describes completely); ^5^ Exercise Planning Scale, possible scores range from 1 (does not describe) to 5 (describes completely); ^6^ Self-efficacy for Walking Scale, possible scores range from 0 (not at all confident) to 100 (extremely confident). * Denotes statistical significance at *p* < 0.01. ^+^ For leisure-time PA in the intervention group, the “less active” mean is significantly less than the “more active” mean (*p* < 0.001).

## Data Availability

The data presented in this study are available on request from the corresponding author.
